# The Diversity of Bacteria Isolated from Antarctic Freshwater Reservoirs Possessing the Ability to Produce Polyhydroxyalkanoates

**DOI:** 10.1007/s00284-014-0629-1

**Published:** 2014-06-18

**Authors:** Slawomir Ciesielski, Dorota Górniak, Justyna Możejko, Aleksander Świątecki, Jakub Grzesiak, Marek Zdanowski

**Affiliations:** 1 Department of Environmental Biotechnology, University of Warmia and Mazury in Olsztyn, ul. Sloneczna 45G, 10-718 Olsztyn, Poland; 2 Department of Microbiology, University of Warmia and Mazury in Olsztyn, ul. Oczapowskiego 1A, 10-719 Olsztyn, Poland; 3 Department of Antarctic Biology, Institute of Biochemistry and Biophysics, Polish Academy of Sciences, ul. Ustrzycka 10, 02-141 Warsaw, Poland

## Abstract

The diversity of polyhydroxyalkanoates-producing bacteria in freshwater reservoirs in the Ecology Glacier foreland, Antarctica, was examined by a cultivation-dependent method. Isolated strains were analyzed phylogenetically by *16S rRNA* gene sequencing, and classified as members of *Alpha*-, *Beta*-, or *Gammaproteobacteria* classes. Polymerase chain reaction was used to detect PHA synthase genes. Potential polyhydroxyalkanoates (PHAs) producers belonging mainly to *Pseudomonas* sp., and *Janthinobacterium* sp. were isolated from all five sampling sites, suggesting that PHA synthesis is a common bacterial feature at pioneer sites. All *Pseudomonas* strains had the genetic potential to synthesize medium-chain-length PHAs, whereas some isolated *Janthinobacterium* strains might produce short-chain-length PHAs or medium-chain-length PHAs. It is the first report revealing that *Janthinobacterium* species could have the potential to produce medium-chain-length PHAs.

## Introduction

A wide variety of taxonomically different groups of microorganisms are known to produce intracellular energy and carbon storage compounds, generally described as polyhydroxyalkanoates (PHAs) [1]. It has been shown that bacterial cells with a higher content of PHAs can survive longer than those with a lower PHAs content, because they can utilize their reserve material longer and more efficiently [7]. Therefore, it was stated that the accumulation of PHAs might increase the survival capabilities of these bacteria in extreme environments or when nutrient availability is poor [8].

PHAs are synthesized by many bacteria and archaea [31] when in environment carbon is available, but other essential nutrients are not. PHAs are commonly divided into two groups based on the number of constituent carbon atoms in their monomer units: short-chain-length (scl-) PHAs and medium-chain-length (mcl-PHAs). The former consists of monomers with 3–5 carbon atoms and the latter consists of monomers with 6–14 carbon atoms. The most commonly produced is polyhydroxybutyrate P(3HB), an scl-PHA. The physical properties of individual PHAs depend on the composition of the monomer units. P(3HB) is highly crystalline, whereas mcl-PHAs are elastic with low melting temperature [32]. These variations in the PHA polymer family create the potential for a vast array of applications [14].

PHAs are accumulated in intracellular granules. The surface of PHA granules is coated with phospholipids and proteins which play a major role in PHA synthesis, degradation, and even in the process of PHA synthesis regulation. The type of PHA produced and accumulated in the granules depends on the metabolic pathway of the particular microorganism. However, independently of the type of synthesized PHAs, the enzyme called PHA synthase always plays the main role [19]. PHA synthases were classified into four classes, according to their substrate specificities and subunits organization. Classes I, III, and IV prefer to synthesize scl-PHA (*phaC* gene), whereas the II class is responsible for mcl-PHA synthesis [20]. Class I of PHA synthase comprises enzyme consisting of single subunit, whereas classes III and IV comprise enzymes that consist of two different types of subunits. The II class of PHA synthase is also built by single subunit but it is coded by two forms of gene that are called *phaC1* and *phaC2* [20]. The nucleotide composition of *phaC1* and *phaC2* genes is very similar but their substrate specificity may differ [10].

Extremophiles inhabiting polar areas are a source of a novel enzymes, which have a great economic potential in many industrial processes, including agricultural, chemical, and pharmaceutical applications [33]. Especially, shallow Antarctic reservoirs influenced by the forces of variable trophic conditions are underexploited sources of microorganisms with biotechnological potential. As a result of selection pressures in these reservoirs, there is a potential for the discovery of novel biochemical pathways leading to PHAs synthesis. Psychrotrophic bacteria, which are adapted to variable temperatures and to other fluctuating conditions such as low nutrient availability and low water quality, may offer important advantages for industrial PHAs production [9]. It is widely accepted that microorganisms from unusual environments provide not only valuable resources for exploiting novel biotechnological processes, but also serve as models for investigating how these biomolecules are stabilized when subjected to changing conditions [12]. The main purpose of this study was to determine whether bacteria inhabiting freshwater reservoirs in glacier forefield areas have the potential to synthesize PHAs.

## Materials and Methods

### Collection Sites

**Fig. 1:**
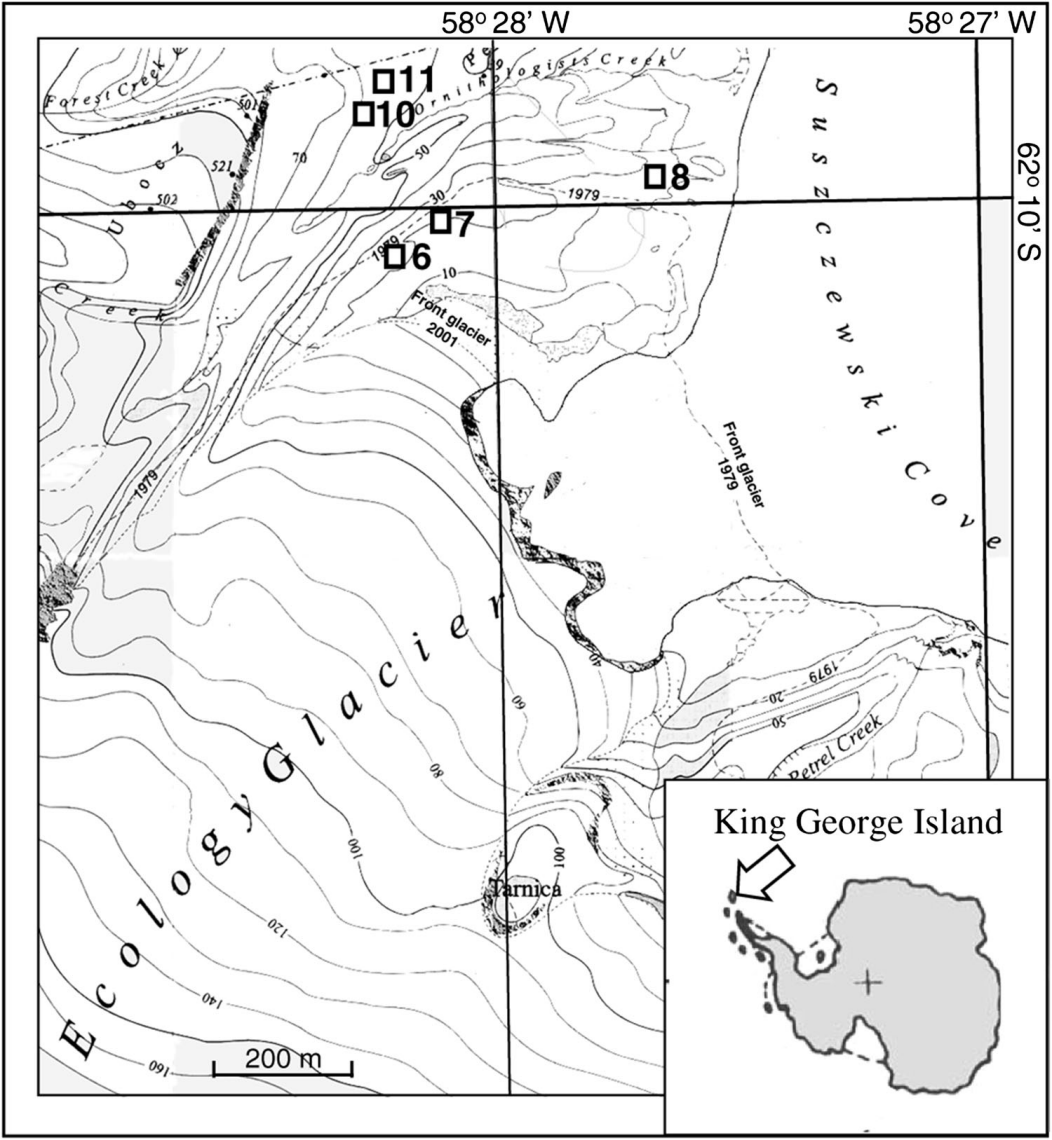
Map showing sampling location in the foreland of Ecology Glacier, King Georg Island, Antarctica. Sampling sites are characterized in Table 1. (Part of Admiralty Bay map modified from Pudełko [17]

Samples of water and mats were collected in summer of 2011 year from five, shallow freshwater reservoirs and one stream. They were located in the foreland of Ecology Glacier near to the Polish Antarctic Station “Arctowski” (King George Island, West Antarctica) (Fig. 1).

Selected reservoirs were different in age, location, and environmental parameters (Table 1). Located near to the front of Ecology Glacier, pond 7 was two years old. This reservoir was located on the ground moraine and was directly supplied with water from the glacier, with no signs of vegetation. Stream 6 is receiving water flowing directly from the glacier. Pond 8 was located away from the front of the glacier, with abundant bloom of microalgae. Reservoirs 10 and 11, which have been noticed there since 1977, were situated on the top of lateral moraine. Both were situated close to each other and connected with small stream (Fig. 1). Samples were taken with a sampler on a bracket into sterile bottles and delivered to the laboratory within 1 hour. Microbial mat samples were collected as cores (1 × 5 cm), and one gram of sample was aseptically weighed and homogenized in 9 ml of sterile cold.(4 °C) physiological saline (0.86 % NaCl) using a vortex. Cultures were obtained by plating 100 μl of a sample onto Antarctic Bacteriological Medium (ABM; pH 7.4) containing: peptone (0.5 %, w/v), yeast extract (0.2 %, w/v), and agar (2 %, w/v) in three replicates. Plates were incubated at 10  °C for 14 days. The well -separated colonies on medium were macroscopically described, photographed, than isolated under binocular, and transferred to slants of ABM in the tubes. After transportation to Poland the microscopic purity evaluation of Gram-stained strains was done.

**Table 1:** Characteristics of sampling sites

Sampling site						
Variables		6	7	8	10	11
GPS position		62°48′26,4 S 058°28′53,9 W	62°48′25,2 S 058°28′50,1 W	62°10′02,3 S 058°2740,9 W	62°09′23,2 S 058°28′20,2 W	62°09′37,0 S 058°28′23,7 W
Elevation. (m a.s.l.)		8	7	9	227	225
Area (m^2^)			266.91	122.30	117.60	123.80
NH_4_-N (mg l^−1^)		0.024 (±0.004)	0.035 (±0.011)	0.426 (±0.071)	0.161 (±0.018)	0.033 (±0.002)
PO_4_-P (mg l^−1^)		0.148 (±0.003)	0.130 (±0.00)	0.120 (±0.042)	0.079 (±0.02)	0.154 (±0.044)
P-tot (mg l^−1^)		0.03 (±0.00)	0.035 (±0.007)	0.050 (±0.028)	0.020 (±0.00)	0.070 (±0.00)
N-tot (mg l^−1^)		0.650 (±0.071)	1.950 (±1.768)	4.100 (±0.00)	1.400 (±0.00)	1.700 (±0.00)
pH (mg l^−1^)		7.6 (±0.5)	7.7 (±0.6)	8.4 (±0.7)	6.5 (±1.2)	7.1 (±0.8)
temp (°C)		2.8 (±1.8)	2.9 (1.6)	5.0 (±0.2)	6.1 (±2.1)	5.8 (±0.6)
O_2_ (mg l^−1^)		11.85 (±0.35)	10.40 (±0.42)	10.86 (±1.22)	11.08 (±0.60)	10.92 (±0.23)
Conductivity (uS/cm)		79.4 (±26.86)	84.7 (±41.37)	166.1 (±76.23)	135.0 (±32.85)	160.3 (±19.38)
Chlorophyll a (μg l^−1^)		0.400	0.417	1.666	0.900	0.900
Pheophytin (μg l^−1^)		2.800	2.791	2.125	3.800	1.800
Total chlorophyll (μg l^−1^)		3.200	3.208	3.791	4.700	2.700
DOC (mg l^−1^)		0.567	0.653	1.135	0.787	0.826

### Molecular Biology Procedures

In order to obtain DNA, cells were scraped from the surface of slants and washed in sterile water. DNA extraction was performed using commercial kit Genomic Mini (A&A Biotechnology) according to the manufacturers’ instructions. The purified DNA was suspended in 50 μl of deionized, DNase free water and stored in −20 °C. The partial *16S rRNA* gene was amplified using the primers *341*: 5′-CCTACGGGAGGCAGCAG -3′ [15] and *16SR*: 5′-TACCTTGTTACGACTTCACCCCA-3′ described previously by Rossau et al. [21]. In order to detect microorganism producing PHAs, PCR was performed with two primer pairs. First one, recognizing both scl- and mcl-PHA synthase genes was elaborated by Romo et al. [22] (*G*-*D*: 5′-GTGCCGCC(GC)(CT) (AG)(GC)ATCAACAAGT-3′; *G1*-*R*: 5′ GTTCCAG(AT)ACAG(GC)A(GT)(AG) TCG AA-3′). The second primers pair (*I*-*179L*: 5′-ACAGATCAACAAGTTCTACATCTT CGAC-3′; *I*-*179R*: 5′-GGTGTTGTCGTTGTTCCAGTAGAGGATGTC-3′) was specific for both genes responsible for mcl-PHA synthesis [25]. PCR was performed in Eppendorf^®^ Mastercycler Gradient (Eppendorf, Germany). The mixtures used for PCR amplification contained 50 ng of extracted DNA, 0.5 μM of each primer, 100 μM of deoxynucleoside triphosphate (Promega, Winsconsin, USA), 1 U of *Taq* DNA polymerase (POLGEN, Poland), and 5 μl of reaction buffer (500 mM KCl, pH 8.5; Triton X-100). PHA synthase gene fragment amplification was carried out using program given by the authors. The temperature program for *16S rRNA* gene amplification was as follows: 94 °C for 5 min; 30 cycles of denaturation at 94 °C for 30 s, annealing at 54 °C for 30 s, extension at 72 °C for 1 min, and single final elongation at 72 °C for 5 min. The PCR amplicons of 16S rDNA and PHA synthase genes were resolved on 1.5 % agarose gels stained with ethidium bromide, and size of PCR products was estimated using molecular weight marker (100 bp, Promega, Winsconsin, USA). The sequencing of 16S rDNA and scl-PHA synthase (*phaC*) PCR products was performed using the same PCR primers as for amplification. Because PCR primers pair *I*-*179L* and *I*-*179R* amplifies both genes coding for mcl-PHA synthase, we use primers *orf2* (5′-CATGACAGCGGCCTGTTCACCTGG-3′ [5] ) and *I*-*179R* [25] using the same conditions as described in the work of Ciesielski et al. [5]. Using this approach, it was possible to amplify and directly sequence a fragment of *phaC* gene about 720 bp long. DNA sequencing was performed using a Perkin Elmer ABI 373 Automated DNA Sequencer (PE Applied Biosystems, Foster City, CA, USA) at the Institute of Biochemistry and Biophysics in Warsaw, Poland. All reactions were run following the manufacturer’s protocols. The nucleotide sequences were submitted to the GenBank database under accession numbers from KF301568 to KF301599.

### Phylogenetic Analysis

The sequences of genes coding for 16S rRNA and PHA synthases were compared with those from the GenBank database using the NCBI blast program. Sequences were aligned using the ClustalW program [30]. The evolutionary distances were inferred using the Neighbor-Joining method [23] with the MEGA5 program [27]. To determine the degree of statistical support for branches in the phylogeny, 1,000 bootstrap replicates of data were analyzed. The gene sequences that were >94.0 % identical to sequences of cultured species in the NCBI database were assigned genus names [26].

### Statistics

A canonical correspondence analysis (CCA) was conducted using a statistical package for Windows v. Canoco 4.5 [29]. The presence of bacteria possessing specific form of PHA synthase genes was analyzed in relation to the environmental background ‘all other data’.

## Results

**Fig. 2:**
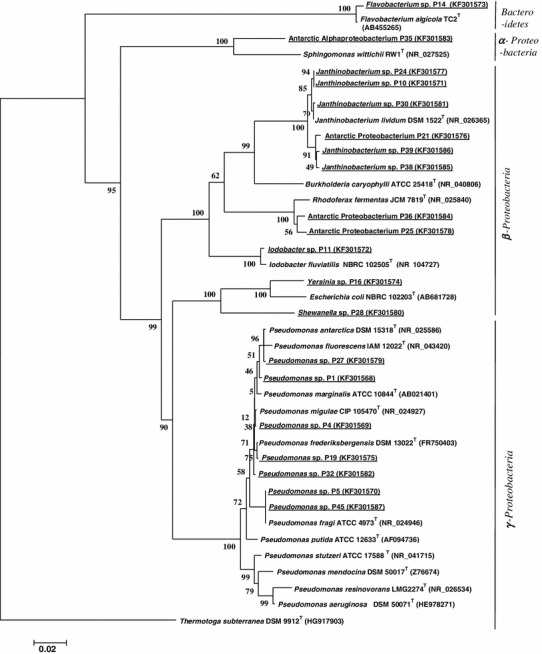
Phylogenetic tree based on 16S rRNA sequences, generated by the neighbor-joining method showing the genetic relationships among bacteria analyzed in this work. *Thermotoga subterranea* (Acc. no HG917903) was selected as the outgroup. Type strains are indicated with superscript T. The numbers on the branches refer to bootstrap values for 1,000 times. Accession numbers of DNA sequences are given in parentheses

A total of 50 bacterial isolates from five ponds and one stream were characterized by sequencing of 1000 bp long fragment of *16S rRNA* gene. Twenty, unique DNA sequences were phylogenetically analyzed altogether with their closest relatives derived from GenBank (Fig. 2). Isolated strains belonged mainly to the phylum of *Proteobacteria* and represent classes *Betaproteobacteria* (11 isolates), *Gammaproteobacteria* (7 isolates), *Alphaproteobacteria* (2 isolates) and only two isolates belonged to *Bacteroidetes* phylum (Fig. 2). Nineteen strains were isolated from ponds and only one (*Pseudomonas* sp. P45) from stream in the foreland of Ecology Glacier. The highest number of strains were derived from pond 10.

The most frequently recovered group of isolates was members of the *Pseudomonas* genus (7 isolates). Among them two strains (*Pseudomonas* sp. P5 and *Pseudomonas* sp. P45) possessed the same DNA sequence of studied *16S rRNA* gene fragments. The second numerous group of isolates belonged to *Janthinobacterium* genus (5 isolates).

In the aim of detection of a microorganism possessing the potential ability of PHAs synthesis, PCR was performed with two primers pairs. The first one (*G*-*D* and *G*-*1R*), is able to recognize both scl- and mcl-PHA synthase genes [22]. The second primers pair (*I*-*179L* and *I*-*179R*) was specific only for mcl-PHA synthase genes [25]. The amplification using primers *G*-*D* and *G*-*1R* gave a positive signal in 12 of the isolates corresponding to six pseudomonads strains, four *Janthinobacterium* strains, and two undefined Betaproteobacteria (P21 and P25). The obtained PCR amplicons possessed the expected length of 551 bp. The amplification with PCR primers designed by Solaiman and co-workers [25] showed that six *Pseudomonas* strains (P1, P4, P19, P27, P32, and P45) and two *Janthinobacterium* strains (P10 and P24) possessed genes responsible for mcl-PHA synthesis. PCR products obtained by employing primer pairs *G*-*D* : *G*-*1R* and *orf2* : *I*-*179R* were directionally sequenced.

**Fig. 3:**
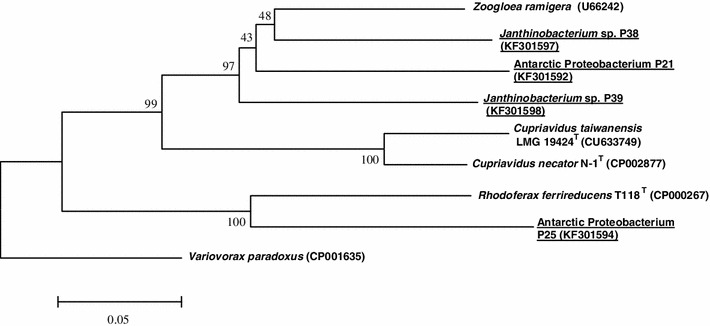
Phylogenetic tree based on *phaC* gene coding for scl-PHA synthase, generated by the neighbor-joining method showing the genetic relationships among bacteria analyzed in this work. *Variovorax paradoxus* (Acc. no CP001635) was selected as the outgroup. Type strains are indicated with superscript T. The numbers on the branches refer to bootstrap values for 1,000 times. Accession numbers of DNA sequences are given in parentheses

**Fig. 4:**
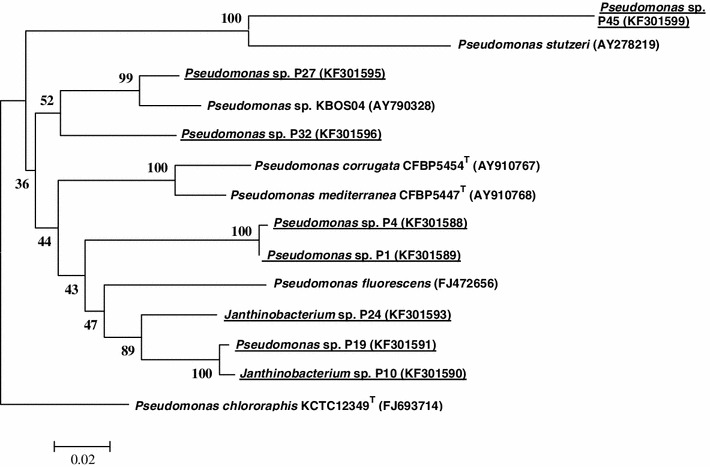
Phylogenetic tree based on *phaC1* gene coding for mcl-PHA synthase, generated by the neighbor-joining method showing the genetic relationships among bacteria analyzed in this work. *Pseudomonas chlororaphis* KCTC12349 (Acc. no FJ693714) was selected as the outgroup. Type strains are indicated with superscript T. The *numbers* on the branches refer to bootstrap values for 1,000 times. Accession numbers of DNA sequences are given in parentheses

The results of DNA sequencing of PCR products obtained using *G*-*D* and *G*-*1R* primers proved that studied DNA of two *Janthinabacterium* strains (P38 and P39), and two undefined Betaproteobacteria strains (P21 and P25) is responsible for scl-PHA synthesis. Nucleotide composition of PCR products amplified with *orf2* : *I*-*179R* primers pair has provided evidence that all are typical for *phaC1* gene coding for synthase responsible for mcl-PHA synthesis. For deeper analysis, all obtained DNA sequences of *phaC* and *phaC1* genes, after division into two group, were phylogenetically examined altogether with their closest relatives found in GenBank. Evolutionary tree presenting genetic distance between *phaC* gene DNA sequences is shown in the Fig. 3. In the *phaC* gene group (scl-PHA) very high similarity (genetic distance 0.236) was revealed between *Janthinobacterium* sp. P39 and Antarctic Proteobacterium P21, inhabiting the same pond 7. Very close to them DNA sequence of *Janthinobacterium* sp. P38 was placed, and all three sequences obtained in this study were accompanied by DNA sequence of *Zooglea ramigera*. The fourth sequence, belonging to Antarctic Proteobacterium P25 was grouped with *Rhodoferax ferrireducens* (genetic distance 0.237) (Fig. 3). The second group, collecting DNA sequences of *phaC1* gene (mcl-PHA), was predominated by members of *Pseudomonas* species. The highest similarity (genetic distance 0.003), was observed between *Pseudomonas* sp. P1 and *Pseudomonas* sp. P4, both inhabiting the mat from lake 10. The next pair, revealing high identity, was composed of *Janthinobacterium* sp. P10 and *Pseudomonas* sp. P19 (genetic distance 0.009) and was collected from different, although neighboring ponds. Surprisingly, these two sequences were grouped together with *Janthinobacterium* sp. P24 sequence that came also from mat of pond 10. The rest of studied *phaC1* gene sequences were dispersed among sequences derived from GenBank. The most diverged *phaC1* gene sequence belonged to *Pseudomonas* sp. P45 that came from the glacier outlet stream (Fig. 4). Bacterial strains possessing genes *phaC* and *phaC1* are characterized in Table 2.

**Table 2:** Molecular identification of isolated strains, their nearest neighbors based on *16S rRNA* and *phaC*/*phaC1* genes and their isolation location

Isolate	Putative PHA	Closest relatives based on *16S rDNA* sequence (Accession no/identity %)^*^	Closest cultured based on *phaC*/*phaC1* gene sequence (Accession no/identity %)^*^	Isolation source
*Pseudomonas* sp. P1	Mcl	*Pseudomonas extremaustralis* SY11 (KC790323/99)	*Pseudomonas fluorescens* Pf0-1 (CP000094/91)	Mat (pond 10)
*Pseudomonas* sp. P4	Mcl	*Pseudomonas frederiksbergensis* (JF343187/100)	*Pseudomonas fluorescens* Pf0-1 (CP000094/90)	Mat (pond 10)
*Pseudomonas* sp. P19	Mcl	*Pseudomonas putida* C14 (JN228297/99)	*Pseudomonas fluorescens* BM07 (FJ472656/91)	Water (pond 11)
*Pseudomonas* sp. P27	Mcl	*Pseudomonas antarctica* (HE586386/99)	*Pseudomonas fluorescens* SBW25 (AM181176/95)	Water (pond 8)
*Pseudomonas* sp. P32	Mcl	*Pseudomonas putida* (HE586397/99)	*Pseudomonas* sp. KBOS04 (AY790328/92)	Water (pond 8)
*Pseudomonas* sp. P45	Mcl	*Pseudomonas fragi* (AB685634/100)	*Pseudomonas stutzeri* 1317 (AY278219/84)	Stream 6
*Janthinobacterium* sp. P10	Mcl	*Janthinobacterium lividum* (HQ824865/100)	*Pseudomonas extremaustralis* (FN435843/90)	Water (pond 10)
*Janthinobacterium* sp. P24	Mcl	*Janthinobacterium lividum* (HQ824864/100)	*Pseudomonas fluorescens* BM07 (FJ472656/92)	Mat (pond 10)
Antarctic Betaproteobacterium P21	Scl	*Burkholderia* sp. Era35(JQ977167/99)	*Zooglea ramigera* (U66242/83)	Water (pond 7)
Antarctic Betaproteobacterium P25	Scl	*Rhodoferax ferrireducens* (HG003356/99)	*Comamonas testosteroni* CNB-2 (CP001220/81)	Water (pond 10)
*Janthinobacterium* sp. P38	Scl	*Janthinobacterium* sp. SMN 33.6 (JX624164/99)	*Zooglea ramigera* (U66242/83)	Water (pond 10)
*Janthinobacterium* sp. P39	Scl	*Janthinobacteriu*m sp. TMT4-26-6 (JX949991/99)	*Zooglea ramigera* (U66242/83)	Water (pond 7)
*Pseudomonas* sp. P5	-	*Pseudomonas fragii* (AB685683/99)	-	Water (pond 8)
*Iodobacter* sp P11	-	*Iodobacter fluviatilis* (KC213858/100)	-	Water (pond 7)
*Flavobacterium* sp. P14	-	*Flavobacterium algicola* (AB455265/99)	-	Water (pond 10)
*Yersinia* sp. P16	-	*Yersinia intermedia* (NR_027545/99)	-	Water (pond 7)
*Shewanella* sp. P28	-	*Shewanella putrefaciens* (KC607503/99)	-	Water (pond 8)
*Janthinobacterium* sp. P30	-	*Janthinobacterium lividum* (HQ824941/99)	-	Water (pond 7)
Antarctic Alphaproteobacterium P35	-	*Sphingomonadaceae* bacterium N (DQ497241/99)	-	Water (pond 7)
Antarctic Betaproteobacterium P36	-	*Rhodoferax ferrireducens* (NR_074760/99)	-	Water (pond 8)

* Only cultured strains were considered during BLAST searching

**Fig. 5:**
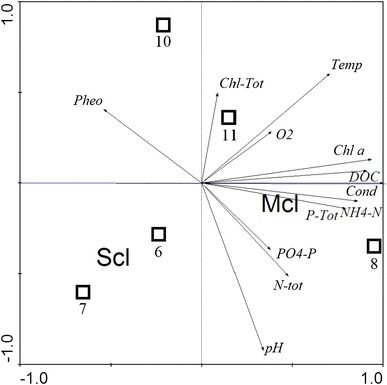
Canonical correspondence analysis showing relation between the presence of bacteria possessing specific form of PHA synthase genes and environmental data of sampling sites

The statistical analysis of principal components (CCA) showed correlation between the presence of bacteria possessing particular PHA synthase gene and environmental conditions. Bacteria having genetic potential to synthesize scl-PHA inhabited reservoirs with rather low trophy characterized by low levels of total phosphorus and high amounts of pheophytins (Fig. 5).

## Discussion

In the ponds that were studied, more than half of the isolated strains possessed genes responsible for PHAs synthesis. Among them were mainly microbes belonging to the *Pseudomonas* genus (six strains) and *Janthinobacterium* genus (four strains). Our results are in concordance with results of Goh and Tan [8]. They isolated from Antarctic soil strains of bacteria having the ability to produce PHAs, among them twelve were belonging to *Pseudomonas* spp. and three to *Janthinobacterium* genus. The presence of *Janthinobacterium* spp. in Antarctic waters has previously been discussed [28]. Shivaji et al. [24] showed that strains of this genus isolated from Antarctica could be divided into two groups; the first is “*Jantinobacterium lividum* group,” whereas the second was called “atypical *J. lividum* group.” Our analysis also indicates that isolated *Janthinobacterium* strains were divided into two groups on the base of gene coding for *16S rRNA* (Fig. 2). Like *Janthinobacterium* genus members, bacteria belonging to *Pseudomonas* spp. are often isolated from cold environments and are known for their metabolic versatility and high genome plasticity [18]. The possibility of PHAs accumulation and high tolerance to cold and oxidative stress is common features of many *Pseudomonas* species. Therefore, it is suggested that PHA metabolism in Antarctic strains could be an adaptation mechanism that is necessary to withstand hard conditions in polar regions [2].

The high number of isolates possessing genes responsible for PHAs synthesis suggests that Antarctic bacteria evolved, or obtained in another way, this metabolic ability to endure the extreme conditions typical for polar regions. Accordingly to our results, isolated strains possessed genes responsible for scl- or mcl-PHA synthesis. It is worth pointing out that bacteria having *phaC1* genes were isolated both from mats and water, and those having *phaC* gene were only planktonic. It has previously been reported that survival in harsh conditions can be increased by belonging to a complex communities like microbial mats [16]. Recent studies have evaluated the potential of microbial mats as high-level PHA production systems under natural conditions, and as a source of bacterial PHA producers [3, 13]. Accordingly to these studies, microorganisms inhabiting microbial mats from marine coastal zones are able to synthesize and accumulate high quantities of PHAs. Our results also show that microbial mats created in Antarctic ponds could be a potential source of PHA producing bacteria.

Our comparative analysis of DNA sequences of *16S rDNA* and *phaC*/*phaC1* genes suggested that some strains possess PHA synthesis genes that might have not been obtained by gradual evolution. The best example are isolates belonging to *Janthinobacterium* spp. Among four members of this genus, possessing genes responsible for PHAs synthesis, two of them had *phaC* genes, whereas two had *phaC1* genes. The *phaC* gene of *Janthinobacterium* spp. P38 and P39 was similar to a variant of this gene previously detected only in *Zooglea ramigera*, whereas the *phaC1* genes of *Janthinobacterium* sp. P10 and P24 were most similar to genes of *Pseudomonas fluorescences.* The genes responsible for scl-PHA synthesis differ so much from those responsible for mcl-PHA synthesis that their presence in these closely related bacteria is most likely not the result of gradual evolution, but rather of horizontal gene transfer. It is interesting that isolates that had obtained these particular types of genes were divided into two subgroups on the basis of *16S rDNA* sequences (Fig. 2). It is possible that this evolutionary separation is related to some physiological differences that make a particular PHA operon useful for these bacteria. Previously, only scl-PHA producing *Janthinobacterium* spp. have been reported [8], and ours is the first report of potential mcl-PHA synthesis by members of this genus.

We found genes coding for mcl-PHA synthase mainly in *Pseudomonas* species. *16S rDNA* gene analysis showed that the taxonomic position of each of the six *Pseudomonas* isolates having the *phaC1* gene was different (Table 2). Whereas, variation between *phaC1* genes was lower, closest DNA sequences of *phaC1* gene of examined strains obtained by BLAST searching belonged only to three genera. The DNA sequences of P1, P4, and P19 were closest to the sequence of *Pseudomonas fluorescens*, a well-known mcl-PHA producer [6]. The structure of the *phaC1* gene of *Pseudomonas* sp. P27 was most similar to the gene of *Pseudomonas* sp. KBOS 04 isolated from activated sludge by Ciesielski et al. [4]. In the same study, the same PHA operon was found in both cells of *Pseudomonas* sp. KBOS04 and of *Comamonas testosteroni*. This observation may suggest that these PHA operons have been horizontally transferred between cells. *Pseudomonas* sp. P45, inhabiting a glacial melt water stream, possessed a *phaC1* gene most similar to that of *Pseudomonas stutzeri*, although the similarity was only 84 %. It should be underlined that the nucleotide composition of the *Pseudomonas* sp. P45 *16S rRNA* gene was most similar to *P. fragi* and identical to the DNA sequence of the *Pseudomonas* sp. P5 isolated from pond 8. In contrast to *Pseudomonas* sp. P45, in cells of strain P5, genes responsible for PHA synthesis were not detected. In general, comparative analysis of *16S rRNA* and *phaC*/*phaC1* genes revealed that isolated bacteria gain the genetic ability to synthesize PHA rather as a result of horizontal gene transfer than by gradual evolution. Furthermore, it seems that some PHA operons are favored during this unusual ecological event.

Bacteria possessing PHA synthase coding genes were detected in each of the studied location suggesting that PHAs accumulation could increase the survival capabilities of microorganisms in this extreme environment. Bacteria having genes responsible for both scl- and mcl-PHAs synthesis were found, but those able of scl-PHA synthesis were discovered only in ponds 7 and 10. These two reservoirs characterized with high amounts of pheophytin and total chlorophyll. The relation between the presence of scl-PHA producing bacteria and environmental conditions is supported by CCA (Fig. 5). The highest number of different forms of *phaC*/*phaC1* genes was founded in pond 10, which was characterized by low pH (6.5), highest level of pheophytin (3.800 μg l^−1^), and total chlorophyll (4.700 μg l^−1^), and with the highest temperature measured (6.1 °C). Only one strain having *phaC1* gene was isolated from pond 11 (*Pseudomonas* sp. P19) and what is interesting similar form of gene was found in cells of *Janthinobacterium* sp. P10 inhabiting pond 10 (Fig. 1). Ponds 10 and 11 are connected with the stream and it is likely, that this form of *phaC1* was channeled from pond 11 to pond 10. A very distant form of *phaC1* gene was found in the mat taken from the stream 6, it might release from the fact that environmental conditions in the streams are different from those in ponds. The spatial distribution of microorganisms possessing particular form of genes in the area of glacier forefield results mainly from glacier melting. The flowing water carries not only nutrients and minerals, but also transfers microorganisms having accelerated ability to colonize new territories. This group can include bacteria having ability to utilize PHAs produced by themselves or other members of microbial community [11]. It seems that bacteria having genetic ability to store and degrade PHAs can work as a pioneers during a succession process. Thus, glacier forefields provide a unique opportunity as a natural laboratory to study the succession of microorganisms.

To sum up, our study shows that many microorganisms inhabiting freshwater lakes charged with glacier meltwater possess the genes responsible for PHA synthesis. Most of the strains possessing PHA synthase genes belonged to *Pseudomonas* and *Janthinobacterium* species. All isolated *Pseudomonas* species had the *phaC1* gene responsible for mcl-PHA synthesis, whereas *Janthinobacterium* isolates had *phaC* and *phaC1* genes used for both scl- and mcl-PHA synthesis. To our knowledge, this is the first evidence that *Janthinobacterium* spp. have the potential to synthesize mcl-PHAs. Another interesting observation is the lack of correspondence between the evolutionary history of the *16S rDNA* genes and those coding for PHA synthases. Phylogenetic analyses of the *phaC* and *phaC1* genes, especially in *Janthinobacterium* sp., suggest that these genes could have been acquired by horizontal gene transfer. The obtained results allow us to speculate that the possession of these genes enabling PHA synthesis may improve the fitness and survival of bacteria in harsh conditions. Additionally, the presented results show that microorganisms inhabiting extreme environments should be considered as potential producers of PHAs.
